# Introducing “In Focus: Basic Research to Broader Impacts”

**DOI:** 10.1021/acscentsci.6c00263

**Published:** 2026-04-22

**Authors:** Kirk S. Schanze, Seth M. Cohen

As an open access journal committed to serving the broad scientific community, *ACS Central Science* continues to publish article formats that highlight not only scientific advances but also the pathways through which those advances generate wider societal benefit. Our existing In Focus series was created to provide a platform for resources and perspectives of broad interest and significance: articles that connect communities, accelerate progress, and illuminate work that has impacts beyond a single scientific subdiscipline.

We now extend this vision with a new thematic series, “In Focus: Basic Research to Broader Impacts,” inspired by contributions such as the recent article (https://doi.org/10.1021/acscentsci.6c00004) detailing how fundamental discoveries in nuclear magnetic resonance translated into transformative tools for chemistry, medicine, and industry. This article traces the path from the earliest curiosity-driven investigations of nuclear spin, to the development of nuclear magnetic resonance spectroscopy, to the emergence of magnetic resonance imaging as a multibillion dollar global technology. It exemplifies a theme we aim to highlight: Fundamental research, often carried out by small groups and supported by diverse funding sources, can ultimately yield breakthroughs with profound societal impact.

This new In Focus series will highlight similarly compelling research to application stories: concise, accessible perspectives that trace research from fundamental insight into real world application. We welcome articles that reveal how basic discoveries lead to new methodologies, industries, public health tools, sustainability practices, or educational innovations. Our goal is to inspire the scientific community and the broader public by underscoring the essential role of fundamental science in driving long-term progress.

We invite authors to propose topics for future installments of this series. Ideal proposals should describe:

- A foundational scientific discovery or conceptual advance;
- The pathway by which it enabled technologies, applications, or societal benefits;
- Insight into the collaborations, tools, or paradigm shifts that accelerated impact; and
- The broader implications for future research directions.

Proposals may include a provisional title, a brief (150–200 word) description of scope, and suggested authorship. We enthusiastically welcome contributions spanning all areas of the chemical sciences and allied fields.

Looking ahead, we hope that In Focus: Basic Research to Broader Impacts will continue the tradition of informing, inspiring, and connecting our community while reminding us that today’s curiosity-driven experiments may become tomorrow’s transformative technologies.

